# On the road to diploidization? Homoeolog loss in independently formed populations of the allopolyploid *Tragopogon miscellus *(Asteraceae)

**DOI:** 10.1186/1471-2229-9-80

**Published:** 2009-06-27

**Authors:** Jennifer A Tate, Prashant Joshi, Kerry A Soltis, Pamela S Soltis, Douglas E Soltis

**Affiliations:** 1 Institute of Molecular BioSciences, Massey University, Palmerston North, New Zealand; 2 Department of Biology, University of Florida, Gainesville, Florida, USA; 3 Florida Museum of Natural History, University of Florida, Gainesville, Florida, USA; 4 Genetics Institute, University of Florida, Gainesville, Florida, USA

## Abstract

**Background:**

Polyploidy (whole-genome duplication) is an important speciation mechanism, particularly in plants. Gene loss, silencing, and the formation of novel gene complexes are some of the consequences that the new polyploid genome may experience. Despite the recurrent nature of polyploidy, little is known about the genomic outcome of independent polyploidization events. Here, we analyze the fate of genes duplicated by polyploidy (homoeologs) in multiple individuals from ten natural populations of *Tragopogon miscellus *(Asteraceae), all of which formed independently from *T. dubius *and *T. pratensis *less than 80 years ago.

**Results:**

Of the 13 loci analyzed in 84 *T. miscellus *individuals, 11 showed loss of at least one parental homoeolog in the young allopolyploids. Two loci were retained in duplicate for all polyploid individuals included in this study. Nearly half (48%) of the individuals examined lost a homoeolog of at least one locus, with several individuals showing loss at more than one locus. Patterns of loss were stochastic among individuals from the independently formed populations, except that the *T. dubius *copy was lost twice as often as *T. pratensis*.

**Conclusion:**

This study represents the most extensive survey of the fate of genes duplicated by allopolyploidy in individuals from natural populations. Our results indicate that the road to genome downsizing and ultimate genetic diploidization may occur quickly through homoeolog loss, but with some genes consistently maintained as duplicates. Other genes consistently show evidence of homoeolog loss, suggesting repetitive aspects to polyploid genome evolution.

## Background

Allopolyploidy combines the processes of hybridization with genome doubling, and together, these provide a potential avenue for instantaneous speciation [1-3]. Whole-genome sequencing efforts have revolutionized our thinking about the significance of polyploidy, as it is clear that paleopolyploidy is a common phenomenon. Ancient whole-genome duplications have been detected in many eukaryotic lineages, including angiosperms, vertebrates, and yeast [4-12]. Polyploidy has been particularly prevalent in flowering plants, where previous estimates indicated that 30–70% of angiosperm species had polyploidy in their ancestry [reviewed in [13]]. In the last decade, the view of polyploidy in angiosperms has changed, and it is now appreciated that perhaps all angiosperm lineages have experienced at least one round of polyploidy, with many lineages undergoing two or more such episodes [14-18]. On more recent timescales, molecular data have also revealed that most extant polyploid plant species have formed recurrently [1,19-28]. In fact, very few examples of a single unambiguous origin of a polyploid species have been documented; these include peanut, *Arachis hypogaea*, the salt marsh grass *Spartina anglica*, and *Arabidopsis suecica *[29-32].

Following allopolyploidization, several evolutionary outcomes are possible for the genes duplicated by polyploidy (homoeologs). Both copies may be retained in the polyploid and remain functional, one copy may accumulate mutations and either diverge in function or become silenced, or one copy may be physically lost [8,33,34]. The fate of these duplicated gene pairs seems to vary depending on the system under investigation and the loci involved [35-41]. Over longer evolutionary timeframes, gene loss, genome downsizing, and, ultimately, genetic 'diploidization' appear to be common phenomena [8,42-45]. Homoeologous recombination appears to play an important role in the loss of small genomic fragments during the early stages of polyploid formation [46-50], which contributes to gene loss and genome downsizing in allopolyploids [39,43,51,52]. Wolfe (2001) pointed out that within a species, some loci may remain 'tetraploid', while others are diploidized; evidence from whole-genome analyses supports this idea [e.g., [36,40]]. Although polyploidy is clearly a recurrent process on both recent and ancient timescales, we know very little about the evolutionary fate of genes duplicated by polyploidy in independently formed allopolyploid populations. Specifically, are homoeologs consistently retained or lost in a repeated manner among individuals from independently formed polyploid populations?

**Figure 1:**
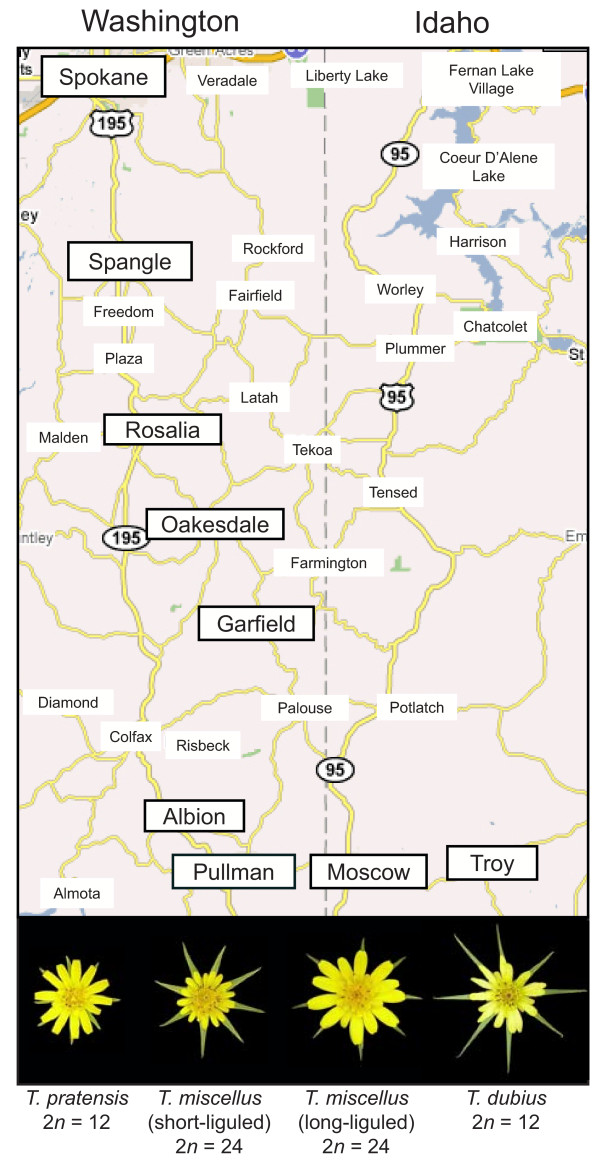
***Tragopogon *populations sampled**. Populations of *Tragopogon *sampled (boxed) in the United States and representative capitula of the diploid (*T. dubius *and *T. pratensis*) and allotetraploid (*T. miscellus*) species. Map modified from Google Maps.

The allopolyploids *Tragopogon mirus *and *T. miscellus *(Asteraceae) are textbook examples of speciation following polyploidy and provide an ideal system to investigate the evolutionary fate of duplicated genes in independently formed populations. These allopolyploids formed recently in the Palouse region of the western United States (eastern Washington and adjacent Idaho) following the introduction of three diploid species (*T. dubius, T. porrifolius*, and *T. pratensis*) from Europe in the early 1900s [53]. *Tragopogon mirus *formed independently several times from *T. dubius *and *T. porrifolius*, while *T. miscellus *formed multiple times from *T. dubius *and *T. pratensis *[53-57]. Only *T. miscellus *has formed reciprocally in nature, and these reciprocally formed individuals can be distinguished morphologically. The 'short-liguled' form has *T. pratensis *as the maternal progenitor, while the 'long-liguled' form has *T. dubius *as the maternal parent (Figure 1). Today, only one long-liguled population exists (in Pullman, Washington; Figure 1); all other populations of *T. miscellus *are short-liguled [58]. Molecular data have confirmed that the populations of *T. mirus *and *T. miscellus *in the Palouse have arisen independently (reviewed in Soltis *et al. *2004; Symonds *et al. *in prep.).

In this study, we examine ten populations of *T. miscellus *for 13 loci to investigate the fate of homoeologous loci in natural populations of *Tragopogon miscellus*. Our previous study [59] revealed that *T. miscellus *individuals from two populations (one each of short- and long-liguled forms) had experienced loss of one parental homoeolog for seven of ten loci examined. This loss was not fixed within or between populations, nor was homoeolog loss 'fixed' for any particular locus examined. Three of these loci were retained in duplicate for all individuals examined. Another recent study has also demonstrated loss of homoeologous loci for five *T. miscellus *populations for a different set of ten genes [60]. In addition to loss, homoeolog silencing has also occurred in these recent allopolyploids [59,60]. Because multiple origins typify most allopolyploid species [27], we extended these previous studies of *T. miscellus *to examine the extent to which parental homoeologs might be lost from individuals from several natural populations and to assess if recurrent patterns of homoeolog loss or retention could be detected in these independently formed populations.

## Results

### Genomic CAPS analyses

**Figure 2:**
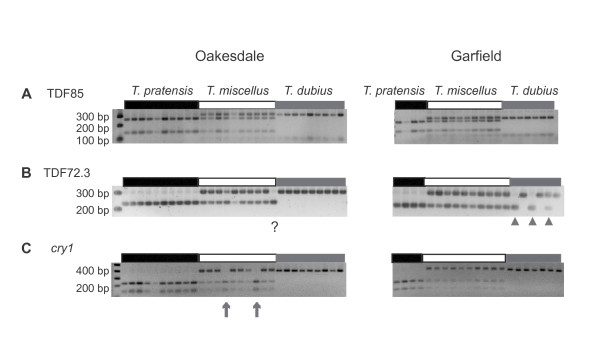
**Loss and retention of homoeologous loci in *Tragopogon miscellus***. Representative genomic CAPS results for three loci from two populations of *Tragopogon miscellus*. An arrow indicates a loss, and an arrowhead indicates allelic variation. **A**. TDF85 showed no losses in any of the populations examined. **B**. Allelic variation was present in *T. dubius *from Garfield for TDF72.3. A 'missing' fragment in *T. miscellus *from Oakesdale may be interpreted as a loss, or the pattern may result from the polyploid individual arising from a *T. dubius *individual with an allele that is similar in its digest pattern to *T. pratensis*. **C**. *cry1 *showed loss in some individuals and some populations, but not others.

Two hundred individuals (83 *T. dubius*, 33 *T. pratensis*, and 84 *T. miscellus*) from 10 populations (Table 1) were screened for 13 markers Table 2). Variation in the restriction digestion patterns of *Tragopogon dubius *was evident for a single marker (TDF72.3) (Figure 2). No variation was observed in *T. pratensis *based on the present sampling. Two individuals, each grown from a seed collected from a *T. dubius *plant in the field (one each from Troy and Albion), apparently were hybrids, as the individuals possessed both *T. pratensis *and *T. dubius *fragment patterns in the genomic restriction digests for all markers screened (data not shown). Because *T. pratensis *does not occur in either locality, these individuals likely represent hybrids between *T. miscellus *and *T. dubius*.

**Table 1:** Populations of Tragopogon analyzed. Populations are ordered geographically from north to south.

Population	Species	Population ID*	Number of individuals
Spokane, WA	*T. pratensis*	--	--
	*T. miscellus*	2664	7
	*T. miscellus*	2617	8
	*T. dubius*	2665	7
Spangle, WA	*T. pratensis*	2692	9
	*T. miscellus*	2693	10
	*T. dubius*	2616	10
Rosalia, WA	*T. pratensis*	--	--
	*T. miscellus*	2667	2
	*T. dubius*	2666	11
Oakesdale, WA	*T. pratensis*	2672	10
	*T. miscellus*	2671	10
	*T. dubius*	2670	9
Garfield, WA	*T. pratensis*	2689	4
	*T. miscellus*	2688	10
	*T. dubius*	2687	7
Albion, WA	*T. pratensis*	--	--
	*T. miscellus*	2625	8
	*T. dubius*	2691	10
Pullman, WA**	*T. pratensis*	--	--
	*T. miscellus*	2605	10
	*T. dubius*	2613	10
Moscow, ID	*T. pratensis*	2608	10
	*T. miscellus*	2604	10
	*T. dubius*	--	--
Troy, ID	*T. pratensis*	--	--
	*T. miscellus*	2682	9
	*T. dubius*	2683	14
	*T. dubius*	2686	5

* Soltis and Soltis collection numbers; **long-liguled population

**Table 2:** Loci analyzed.

Locus ID	Gene abbreviation	Putative protein/gene
TDF7	CKINS	Casein kinase
TDF17.4	UBQ	Polyubiquitin
TDF36.3	THIOR	Thioredoxin M-type 1
TDF44	LTR2	Leucine-rich repeat transmembrane protein kinase
TDF46	PP2C	Protein phosphatase 2C family protein
TDF62	AUX	Auxin conjugate hydrolase
TDF72.3	ADG	Putative adenine-DNA glycosylase
TDF74	TDRC	Transducin family protein
TDF85	BFRUCT	β-fructosidase
TDF90	GTPB	Small GTP-binding protein
TDF27.10	PSBO	Oxygen-evolving enhancer
cry1	cry1	Cryptochrome 1
nrDNA	nrDNA	Nuclear ribosomal DNA

Combining the new data generated here with data from Tate *et al. *(2006), for the 13 loci examined, 11 showed loss of a homoeolog in at least one of the *T. miscellus *individuals surveyed (See Additional file 1; Figure 2). For two genes (TDF46 and TDF85), both parental homoeologs were retained in all individuals examined. Some genes showed loss more frequently than others. For example, a *T. pratensis *homoeolog of TDF17.4 was lost in only one individual, while for TDF90, 18 individuals lost either the *T. dubius *or *T. pratensis *copy. For the genes that showed losses, 20 losses were from the *T. pratensis *genome, while 40 were from the *T. dubius *genome (χ^2 ^= 6.667, df = 1, *P *< 0.001). Considering the genic patterns across populations, none of the genes showed loss in every population. One gene, TDF36.3, showed loss in at least one individual from all but one population (Troy).

Forty of the 84 *T. miscellus *individuals surveyed showed loss of a homoeolog for at least one locus, with 15 of these showing loss for multiple loci (See Additional file 1). For example, individual 2693-14 from Spangle lost the *T. dubius *homoeolog for both TDF7 and TDF90 and lost the *T. pratensis *homoeolog for TDF62; individual 2625-3 from Albion lost the *T. pratensis *copy for TDF7, TDF44, TDF74, TDF36.3, TDF27.10, and *cry1*. For individuals that lost a homoeolog at more than one locus, the same parental homoeolog was lost more often than different homoeologs (i.e., 11 individuals lost homoeologs from the same parent, while four individuals lost alternative homoeologs). Of those that lost the same parental homoeolog, nine cases were losses of *T. dubius*, while two were losses of *T. pratensis*. Considering all populations, regardless of the parental origin, the loss of one homoeolog was the most common scenario (25 cases), followed by homoeolog losses at two loci in eight individuals, three loci in six individuals, and six losses in one plant (individual 2625-3 from Albion, mentioned above). For these multiple losses, no clear pattern emerged (i.e., when two or more loci were lost from multiple individuals, they were not the same pairs of loci).

At the population level, differences in the number of losses were also evident, but without a clear genomic, genic, or geographical pattern (See Additional file 1). The Albion and Moscow populations showed the greatest number of total homoeolog losses (12 losses in three and seven individuals, respectively), followed by Oakesdale (nine losses in five individuals), Pullman (eight losses in six individuals), Spokane-2617 (six losses in five individuals), Garfield (six losses in five individuals), Spangle (six losses in four individuals), Troy (four losses in two individuals), Rosalia (one), and Spokane-2664 (one). The number of individuals (within a population) that showed the same pattern of loss varied by population. The Spokane-2664, Spangle, Rosalia, Garfield, Moscow, and Troy populations did not have any individuals that shared patterns of loss. Spokane-2617 and Pullman each had three individuals with shared patterns, while Oakesdale and Albion each had two individuals. Shared losses among individuals within a population may represent inheritance of a loss that occurred in a common ancestor.

## Discussion

### Homoeolog loss in independently formed populations

Our extended survey of 13 loci for ten populations of *Tragopogon miscellus *indicates that some genes are maintained in duplicate in all populations, while others show loss among some individuals from the independently formed populations. This result is consistent with our previous finding of loss in two populations (Moscow and Pullman) for ten of these same genes [59]. Although homoeolog loss is not unique to *Tragopogon*, the present study represents the largest survey of individuals from natural populations conducted thus far. Homoeolog loss appears to be a common phenomenon in polyploids and may occur rapidly following their formation. For example, synthetic polyploids of wheat [47] and *Brassica *[46,48,61] show loss of homoeologous loci in early generations. In *Tragopogon*, we have not detected loss in F_1 _hybrids or first-generation synthetic polyploids [59,60]. Thus, homoeologous loss does not appear to occur instantaneously upon hybridization or polyploidization in *Tragopogon*, at least based on the loci examined thus far.

Given that genome downsizing and other processes may ultimately contribute to genetic 'diploidization' in polyploid organisms [8,43], what impact does homoeolog loss have on recently and independently formed polyploid populations? Our data indicate that homoeolog loss in *Tragopogon miscellus *is stochastic among individuals from polyploid populations that are less than 80 years old (<40 generations as these are biennials). Almost half (48%) of the individuals surveyed here showed loss of a homoeolog for at least one locus, with some populations showing loss more frequently than others. Five of these same populations were examined for a different suite of ten genes by Buggs *et al. *(2009), and a similar result was found. The Moscow population showed the greatest number of losses (eight), followed by Oakesdale (five), Garfield (three), Spangle (one), and Pullman (one). Some of the same individuals were examined here and as part of that study, but again, no clear pattern of loss among individuals and populations could be identified. Given the ecological success of *T. miscellus*, which is widespread in the Palouse and whose range is expanding [58], this loss does not appear to negatively affect the individuals or populations. When Ownbey [53] first described *T. miscellus *and *T. mirus*, he found that fertility (seed set) averaged 52–66% in the natural populations, but with a great deal of variation outside this range among individuals. More recent surveys of the natural populations indicate that fertility (based on pollen stainability) is high, averaging 95–100% (P. Soltis and D. Soltis, unpublished data), which suggests that following their initial formation the polyploid individuals experience some genomic instability, but eventually become more stabilized. Recently, we resynthesized allopolyploids of both *T. miscellus *and *T. mirus *[62]. The initial S_1 _plants exhibited slightly reduced pollen stainability and fruit set; but successful lineages that have survived to the S_2 _generation exhibit high fertility. Through homoeolog loss, perhaps the polyploid individuals from natural populations are still sorting out potential genomic incompatibilities resulting from hybridization and genome doubling [63]. It will be important to follow the synthetic polyploids through successive generations to determine when homoeolog loss occurs and if this loss contributes to increased fertility.

One consistent pattern among the populations that has emerged from the present study is that *T. dubius *homoeologs appear to be lost more often than those of *T. pratensis*, particularly when two or more loci undergo loss, and this difference in losses is statistically significantly different (*P *< 0.001) (See Additional file 1). Combining the data presented here with those from Tate et al. (2006), we find that loss of a *T. dubius *homoeolog represents 67% of the total losses, while loss of the *T. pratensis *copy represents 33% of the total losses. Why the *T. dubius *copy is eliminated more frequently is not known. Significantly, other allopolyploids, including wheat [49,64] and *Brassica *[61], also show biased elimination of one parental genome over the other. The loss of *T. dubius *homoeologs is especially evident for nrDNA (See Additional file 1, Figure 3) and is consistent with previous studies of nrDNA evolution in natural populations of *T. mirus *and *T. miscellus *[65,66]. Matyášek *et al. *(2007) found that although the allopolyploids had fewer *T. dubius *nrDNA copies, these were preferentially expressed over the alternative parental copies (i.e., either *T. porrifolius *or *T. pratensis *for *T. mirus *and *T. miscellus*, respectively). In the present study, we also identified a few individuals that have reduced *T. pratensis *nrDNA copy numbers relative to *T. dubius*. These individuals were from both short- and long-liguled *T. miscellus *populations (See Additional file 1, Figure 3). This bi-directional concerted evolution of nrDNA copies has also been demonstrated in more ancient allopolyploids, such as *Gossypium *(*G. tomentosum*, *G. hirsutum*, *G. darwinii*, *G. barbadense*, *and G. mustelinum*) [67].

**Figure 3:**
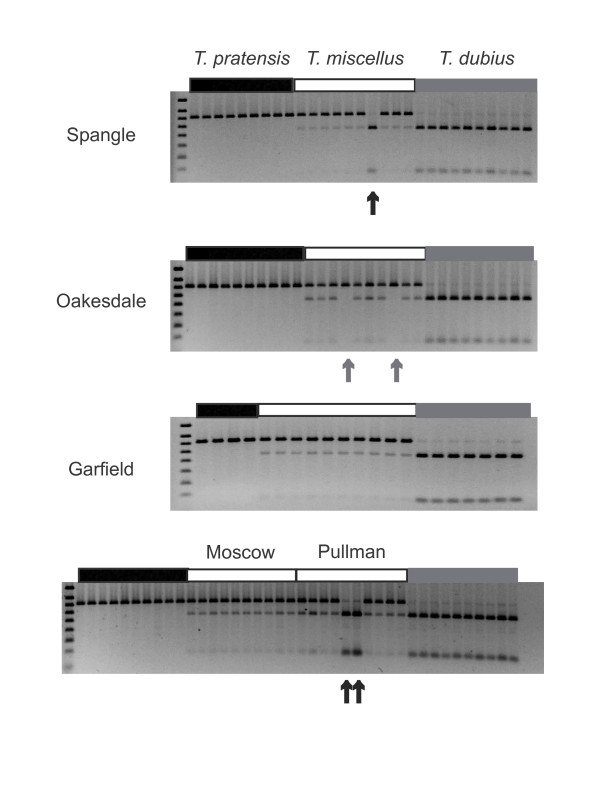
**nrDNA variation in populations of *Tragopogon miscellus***. Most individuals show genomic digest profiles of *T. pratensis *nrDNA copies > *T. dubius *nrDNA copies, although a few individuals show the opposite pattern, and some individuals have lost a parental locus entirely (indicated by an arrow).

The loss or retention of certain classes of genes appears to be a recurrent pattern when ancient whole-genome duplication patterns are examined, although the classes that are retained in duplicate differ depending on the lineage under study [35,36,41,68]. For example, in Asteraceae, Barker *et al. *(2008) found that genes associated with structural components and cellular organization were retained in duplicate, while genes involved with regulatory (e.g., transcription factors) and developmental functions lack duplicates. In *Arabidopsis *(Brassicaceae) and rice (Poaceae), however, genes involved with transcription were retained in duplicate [36]. In *Tragopogon miscellus*, the two genes that were retained in duplicate (TDF46 and TDF85) in all individuals did not fall into the category of significantly enriched (or reduced) when compared to the Barker *et al. *(2008) study. Similarly, the genes that were lost did not match gene ontology (GO) slim categories that were significantly either underrepresented or enriched. TDF46 is a putative protein phosphatase 2C family protein that functions in the plasma membrane, and TDF85 is a putative β-fructosidase that acts in the vacuole. As additional genomic resources are developed for *Tragopogon *and these genes are analyzed in the polyploid species, it will be imperative to determine whether certain gene classes are consistently lost or retained following allopolyploidization.

### Mechanism for homoeolog loss

Studies of *Brassica *[46,50,61] allopolyploids have revealed a significant role for homoeologous recombination in DNA loss, although this process does not appear to affect wheat allopolyploids [38]. A recent karyological study using fluorescent and genomic *in situ *hybridization (FISH, GISH) of natural and synthetic *Tragopogon *allopolyploids identified extensive chromosomal changes, including monosomy and trisomy, intergenomic translocations, and variation in nrDNA loci [69]. Importantly, the same study [69] showed that some chromosomal changes occurred in the first synthetic generation of *T. mirus *(synthetics of *T. miscellus *have not yet been investigated). Ownbey [53] observed multivalent formation in individuals of *T. mirus *and *T. miscellus *from natural populations and also noted univalents and a ring of four chromosomes in F_1 _hybrids between *T. dubius *and *T. pratensis*. We have also observed frequent multivalent formation in synthetic lineages of *T. mirus *and *T. miscellus *[62]. These prevalent meiotic irregularities suggest a mechanism for the homoeolog losses observed here. That is, through homoeologous recombination in early generations following polyploid formation, genome reshuffling and gene loss could act to stabilize the new polyploid genome [63]. Perhaps in *Tragopogon *a combination of factors acts over time to stabilize the new polyploid genomes. For example, some chromosomal changes could happen immediately following polyploid formation, with homoeolog loss acting gradually over successive generations. The study of additional genes and comparisons with synthetic *T. miscellus *lineages [62] over several generations will be important for establishing the overall pattern of genome change in this system.

## Conclusion

Our survey of 13 homoeologous loci in individuals from ten populations of *Tragopogon miscellus *represents the most extensive survey of the fate of duplicate genes in allopolyploid genomes from independently formed natural populations. In this species, loss of a parental homoeolog has occurred for several loci in individuals from these populations. Some loci are consistently maintained as duplicates in all individuals from these populations. Other genes consistently show evidence of homoeolog loss across populations of independent origin; significantly, the *T. dubius *homoeolog is typically lost. Hence, some aspects of genome evolution appear to have been repeated in these new polyploids. In these young (~40 generations) allopolyploids, genomic incompatibilities may be resolved, in part, through loss of a parental homoeolog for some loci. As polyploidy and genome downsizing are recurrent processes in many lineages, other polyploid groups should be investigated to determine if similar patterns emerge for the loss and retention of genes duplicated by polyploidy.

## Methods

### Plant material and population sampling

Mature fruiting heads of *Tragopogon dubius, T. miscellus*, and *T. pratensis *were collected from multiple individuals from several populations in Washington and Idaho, USA, in July 2005 (Table 1). Seeds were germinated in 11.4 cm pots in a glasshouse at the University of Florida (Gainesville, FL, USA) under standard conditions. Material from Pullman, Washington, and Moscow, Idaho, was utilized from a previous study [59].

In total, we included ten populations of *T. miscellus*, four populations of *T. pratensis*, and nine populations of *T. dubius *(Table 1). Our sampling strategy was intended to survey as many individuals and populations from the Palouse as possible. Similarly, we recognize that sympatric diploid populations may not represent the progenitor genotypes for a particular local polyploid population (although they typically do; Symonds et al. unpublished). Therefore, we wanted to survey as many diploid individuals as possible to screen for potential variation in the loci examined. The number of populations and number of individuals from the diploid populations included in the study differed because of changes in population dynamics since the formation of the *Tragopogon *polyploids [70]. For example, while once locally common, *T. pratensis *has become sparse in the Palouse over the last several decades [58] and is not always found in the vicinity of *T. miscellus *populations (Table 1). Nevertheless, data accumulated from previous studies [23,57] indicate that very little genetic variation exists within and between populations of *T. pratensis *in the Palouse. On the other hand, *T. dubius *is more widely distributed [58] and harbors more genetic variation than does *T. pratensis *[57]. Of the two diploid parents of *T. miscellus*, *T. dubius *is more likely to exhibit variation in the genes examined.

### Genomic CAPS analysis

To determine if parental homoeologs were maintained or lost in the *Tragopogon miscellus *individuals from independently formed populations, we used genomic cleaved amplified polymorphic sequence (CAPS) analysis [71]. Leaf material was harvested from seedlings and DNA extracted following a modified CTAB protocol [72]. For two of the three loci not previously analyzed (*cry1 *and TDF27.10), primers were designed from *Tragopogon dubius *sequences using Primer3 [73]. The *cry1 *sequence originated from an EST library of *T. dubius *(Tdu01-6MS1_D11.e), and the TDF27.10 (TDF stands for transcript-derived fragment) sequence was derived from a previous cDNA-AFLP study [59]. Primer sequences for these two loci were cry1-1F: 5'-AATGGTTCCCAGTTTGACCA-3', cry1-1R: 5-GGCAAAGTTTTACCCGGTTT-3'; TDF27.10F: 5'-CATTCATGCAACCAACCAAG-3', TDF27.10R: 5'-CTTCGGACTTCCTTCAGCAC-3'. These primers were used to amplify genomic DNA from *T. dubius *and *T. pratensis *with the aim of identifying sequence polymorphisms that could distinguish the homoeologs in *T. miscellus*. Genomic amplifications were conducted in a 25 μL volume with 50 ng template, 10× Thermopol buffer (New England Biolabs, Ipswich, MA, USA), 0.4 mM dNTPs, 0.2 μM each primer, and 0.5 unit Taq polymerase (New England Biolabs). Thermal cycling conditions were as follows: 94°C for 2 min, followed by 35 cycles of 94°C for 30 sec, 52–54°C for 30 sec, 72°C for 1 min, and a final 5-min extension at 72°C. Products were separated on a 1.5% agarose gel, stained with ethidium bromide, and visualized by UV on a transilluminator. PCR products were prepared for sequencing by adding 5 units of Exonuclease I (Fermentas, Glen Burnie, MD, USA) and 0.5 unit Shrimp alkaline phosphatase (Fermentas) and treating them at 37°C for 30 min followed by 80°C for 15 min. Cleaned products were separated on an ABI 3770 following the manufacturer's recommendation (Applied Biosystems, Foster City, CA, USA). Sequences were edited in Sequencher version 4.7 (Gene Codes, Ann Arbor, MI, USA) and deposited in GenBank under accession numbers FJ770374–FJ770377. To determine if sequence polymorphisms between the two diploid parental sequences occurred at a restriction site, the sequences were analyzed with dCAPS Finder 2.0 [74]. For *cry1*, the restriction enzyme *AciI *cut the *T. pratensis *382-bp product into two fragments (232 and 150 bp), while the *T. dubius *product remained uncut (385 bp). For TDF27.10, *MseI *cut the *T. pratensis *PCR product into two fragments (216 and 83 bp), and the *T. dubius *product remained uncut at 299 bp. Restriction digestions for both markers were conducted in a 10-μl volume with 1× buffer (New England Biolabs), 1 μL PCR product, 5–10 units of enzyme (New England Biolabs), and 100 μg/ml Bovine Serum Albumin (when required). The reactions were allowed to incubate at the temperature specified by the supplier for three hours. Digested products were separated on a 2% agarose gel, stained with SybrGold (Molecular Probes Inc., Eugene, OR, USA), and visualized on a transilluminator. Once the utility of these markers was established, the remaining individuals of *T. dubius*, *T. miscellus*, and *T. pratensis *were PCR-amplified and digested in the same manner. For nrDNA repeats in *T. miscellus*, genomic CAPS analysis was conducted as described in Kovarík *et al. *[65].

For the previously analyzed markers (TDF7, TDF17.4, TDF36.3, TDF44, TDF46, TDF62, TDF72.3, TDF74, TDF85, and TDF90), genomic amplification and restriction digestion were conducted as described in Tate *et al. *[59]. The Moscow and Pullman populations of *T. miscellus *were the subject of a previous study [59]; those data are combined here with data for three new loci (*cry1*, TDF27.10, and nrDNA).

To verify that the observed homoeolog losses based on CAPS analysis were not the result of a point mutation at the diagnostic restriction site in *T. miscellus *post-polyploid formation, PCR products were sequenced for all individuals of *Tragopogon miscellus *that showed loss of a homoeologous fragment. For a given individual, a homoeolog loss was scored only when the sequence data verified the pattern from the CAPS gel analysis (i.e., no sequence polymorphisms were detected in the chromatogram either at the diagnostic restriction site or at other positions where *T. dubius *and *T. pratensis *differ). These same criteria applied for nrDNA loci. However, when the intensity of the digested parental fragments differed in the CAPS gel, the nrDNA patterns were scored as P > D or D > P to reflect differing copy numbers in the allopolyploid individuals [65,66]. For all loci, when a loss was determined, we assumed that both alleles of a parental homoeolog were lost. In cases where one allele of a homoeolog was lost, CAPS analysis might not detect these losses. Furthermore, identical patterns of loss in individuals from the same population may be the result of shared ancestry. Therefore, total losses from a single population were tabulated as both minimum and maximum number of losses.

## Authors' contributions

JAT designed the study, collected and analyzed genomic CAPS data, and drafted the manuscript. PJ and KAS conducted genomic CAPS analyses. PSS and DES helped to design the study and contributed to drafting the manuscript. All authors read and approved the final manuscript.

## Supplementary Material

Additional file 1 **Summary of homoeolog losses in *Tragopogon miscellus***. A '+' symbol indicates that no losses were detected in a population for a particular gene. 'D' or 'P' following an individual number indicates the parental homoeolog lost (D = *T. dubius*; P = *T. pratensis*) from that individual. Click here for file
