# Supplementary material for: On the road to diploidization? Homoeolog loss in independently formed populations of the allopolyploid Tragopogon miscellus (Asteraceae)
Source: BMC Plant Biol. 2009 Jun 27;9:80. doi: 10.1186/1471-2229-9-80 (PMC2708164; doi:10.1186/1471-2229-9-80)
Supplement: Additional file 1 — Summary of homoeolog losses in Tragopogon miscellus. A '+' symbol indicates that no losses were detected in a population for a particular gene. 'D' or 'P' following an individual number indicates the parental homoeolog lost (D = T. dubius; P = T. pratensis) from that individual. [file 1471-2229-9-80-S1.doc]

Title: Summary of homoeolog losses in *Tragopogon miscellus*.

Description: A ‘+’ symbol indicates that no losses were detected in a population for a particular gene. ‘D’ or ‘P’ following an individual number indicates the parental homoeolog lost (D = *T. dubius*; P = *T. pratensis*) from that individual.

|  | N | TDF46 | TDF85 | TDF17.4 | TDF7 | TDF62 | TDF72.3 | TDF44 | TDF74 | TDF36.3 | TDF90 | TDF27.10 |  |  |
| --- | --- | --- | --- | --- | --- | --- | --- | --- | --- | --- | --- | --- | --- | --- |
| Population |  | PP2C | BFRUCT | UBQ | CKINS | AUX | ADG | LTR2 | TDRC | THIOR | GTPB | PSBO | cry1 | nrDNA** |
| Spokane, WA (2664) | 7 | + | + | + | + | + | + | + | + | 2664-24P | + | + | + | 2664-7D>P |
| Spokane, WA (2617) | 8 | + | + | + | + | + | + | + | 2617-4D | 2617-1D | 2617-1D | 2617-7D  2617-12D  2617-21D | + | P>D |
| Spangle, WA | 10 | + | + | + | 2693-14D | 2693-14P | + | + | 2693-5D | 2693-12D | 2693-14D  2693-15D | + | + | 2693-12D>P |
| Rosalia, WA | 2 | + | + | + | + | + | + | + | + | 2667-1P | + | + | + | P>D |
| Oakesdale, WA | 10 | + | + | + | + | + | + | 2671-6P | + | 2671-8P  2671-10D | 2671-4D  2671-9D | + | 2671-4D  2671-9D | 2671-4D  2671-9D |
| Garfield, WA | 10 | + | + | + | + | 2688-8P | + | + | 2688-10D | 2688-6P | 2688-2D  2688-10D  2688-12P | + | + | P>D |
| Albion, WA | 8 | + | + | 2625-3P | + | + | + | 2625-3P | 2625-3P  2625-9D  2625-10D | 2625-3P | + | 2625-3P  2625-9D  2625-10D | 2625-3P | 2625-2D>P  2625-3D>P  2625-9D  2625-10D |
| Pullman, WA* | 10 | + | + | + | + | + | + | 2605-24D | + | 2605-28D | 2605-13D  2605-14D  2605-24D  2605-28P  2605-29P  2605-42D | + | + | 2605-14D>P  2605-24D>P |
| Moscow, ID* | 10 | + | + | + | 2604-35D | 2604-11D | 2604-15D  2604-22D | 2604-22P | 2604-4P  2604-35D | 2604-20D | 2604-4D  2604-15D  2604-22D  2604-24D | + | + | P>D |
| Troy, ID | 9 | + | + | + | + | 2682-2P | + | 2682-2P | + | + | + | 2682-4D | + | 2682-7D |

* Data for first ten loci were compiled from Tate *et al.* (2006)

** Individuals were scored as P>D unless otherwise noted
